# Native Amazonian Inga Fruits as Sources of Bioactive Compounds with *In Vitro* Antioxidant and Antimicrobial Activity

**DOI:** 10.3390/molecules31193471

**Published:** 2026-09-29

**Authors:** Elena Coyago-Cruz, Gabriela Méndez, Marco Cerna, Edwar Mindiola, Jorge Heredia-Moya

**Affiliations:** 1 Carrera de Ingeniería en Biotecnología, Universidad Politécnica Salesiana, Sede Quito, Campus El Girón, Av. 12 de Octubre N2422 y Wilson, Quito 170143, Ecuador; 2 Centro de Investigación Biomédica (CENBIO), Facultad de Ciencias de la Salud Eugenio Espejo, Universidad UTE, Quito 170527, Ecuador

**Keywords:** Ecuadorian Amazon, functional ingredients, underutilised fruits

## Abstract

The genus *Inga* (Fabaceae), widely distributed across the Neotropics, comprises numerous underexplored native species with potential as functional foods. This study evaluated the physicochemical, mineral, and bioactive characteristics, as well as the in vitro antioxidant and antimicrobial activities, of eight *Inga* species from the Ecuadorian Amazon. Physicochemical parameters were determined; minerals were quantified by atomic absorption spectrophotometry, while vitamin C, organic acids, carotenoids, chlorophylls, and phenolic compounds were analysed by RRLC. Antioxidant activity was assessed using the DPPH and ABTS assays, and minimum inhibitory concentrations were determined by broth microdilution. *Inga velutina* presented 24.0 °Brix, whereas *Inga bachryrhachis* contained 2941.3 mg/100 g DW potassium, 464.9 mg/100 g DW calcium and 7703.7 mg/100 g DW phenolic compounds. *Inga leiocalycina* contained 13.8 mg/100 g DW vitamin C, while *Inga feuilleei* reached 2233.9 mg/100 g DW total organic acids. *Inga capitata* exhibited the highest DPPH and ABTS values, at 4.1 and 5.3 mmol TE/100 g DW, respectively. *Inga densiflora* inhibited *Escherichia coli* and *Streptococcus mutans*, with an MIC of 5.2 mg/mL. PCA explained 62.5% of the total variability. These findings support the selective valorisation of native *Inga* fruits as potential sources of bioactive ingredients.

## 1. Introduction

The genus *Inga* belongs to the family Fabaceae, which is considered the third-largest family of angiosperms after the *Asteraceae* and *Orchidaceae*. It comprises approximately 300 species with a Neotropical distribution ranging from Mexico to northern Argentina, with the greatest diversity found in the Andean region of Peru, Ecuador and Colombia [1,2]. The term Inga or Angá derives from an indigenous word meaning ‘soaked’ or ‘containing seeds’, in reference to the watery, sweet and edible pulp that surrounds the seeds of its fruits. Owing to its many uses, the genus constitutes one of the most important plant resources of the Amazon, where, in addition to forming part of the traditional diet, it plays a significant role in agroforestry systems, the restoration of degraded areas and the traditional medicine practised by various indigenous communities [3,4].

*Inga* species grow mainly in areas near rivers and lakes. Their fruits are elongated, cylindrical green pods containing black seeds covered by a sweet-tasting white pulp. In addition to being eaten fresh, various parts of these species have traditionally been used for medicinal purposes. The fruits are used as an anti-diarrhoeal, whilst the seeds are used to treat cancer. These properties have been linked to the presence of bioactive metabolites that confer high antioxidant activity, including anthocyanins, flavonoids, phenolic acids, catechins, trypsin inhibitors, and saponins [5].

Recent decades of chemical and pharmacological research have shown that the genus *Inga* is an important source of secondary metabolites with biological potential. Studies on leaves, bark and fruits have identified terpenoids, steroids, saponins, nitrogenous compounds, flavonoids, phenolic acids and other specialised metabolites [4]. The phenolic fraction includes flavonoids, anthocyanins, tannins, phenolic acids, and proanthocyanidins. By contrast, triterpenes and phytosterols belong to the terpenoid and steroid classes, respectively, while saponins are glycosides generally containing triterpenoid or steroid aglycones. Nitrogen-containing metabolites derived from *L*-tyrosine, proline and pipecolic acid constitute another chemically distinct group and have been described as chemical markers of the genus [3].

The chemical diversity of the genus has also been demonstrated through the isolation of specific metabolites in different species. In the leaves of *Inga umbellifera*, flavonoids and proanthocyanidins derived from catechin were isolated; in *I. goldmani*, a trimeric proanthocyanidin was identified; in *I. paterno*, trans-4-methoxypipecolic acid was isolated; and in the leaves and bark of *I. edulis*, derivatives of catechin, myricetin, quercetin and terpenes related to stigmasterol were identified [1].

The phytochemical richness described for *Inga* is reflected in the wide variety of biological activities reported for its species. Previous studies have demonstrated antimicrobial activity in *I. fendleriana*, antioxidant activity in *I. edulis* and *I. verna*, and antifungal and antitumour activity in *I. marginata* [4]. Furthermore, the pharmacological properties described for the genus include antioxidant, antimicrobial, insecticidal, antiparasitic, allelopathic, antifungal and antitumour activities, highlighting its importance for both pharmaceutical and agronomic applications [3].

Among the secondary metabolites present in *Inga*, phenolic compounds are among the most important due to their abundance and structural diversity. To date, more than 8000 chemical structures belonging to this family have been described, ranging from simple molecules, such as phenolic acids, to complex polymers such as tannins [1].

In plants, these compounds are involved in various defence mechanisms against ultraviolet radiation and attack by phytopathogenic microorganisms. In food, interest in these compounds has increased considerably as numerous studies have demonstrated their ability to prevent or help alleviate chronic diseases, including cancer, diabetes, cardiovascular diseases and neurodegenerative diseases. Furthermore, they represent a promising alternative to the synthetic antioxidants used by the food industry [6].

Among the species studied, *Inga marginata* is one of the best examples of the genus’s functional potential. This species, widely distributed across Central and South America, produces long, greenish-yellow pods containing numerous seeds surrounded by sweet pulp. Its fruits contain phenolic compounds such as tannins and proanthocyanidins, as well as chemically distinct phytosterols, and triterpenes—metabolites associated with vasodilatory, antithrombotic and anticancer activities. Furthermore, recent research has demonstrated antimicrobial activity in *I. fendleriana* against Gram-positive bacteria and antifungal activity in leaf and bark extracts of *I. laurina*, effects attributed mainly to the presence of phenolic compounds [6].

Despite growing interest in the genus *Inga*, existing knowledge continues to focus on a small number of species and, in many cases, on leaves or bark. In contrast, the physicochemical composition, mineral content, profile of bioactive compounds and functional properties of numerous native species remain virtually unexplored, particularly those found in the Ecuadorian Amazon. This limited information restricts their utilisation as food resources and hinders the exploitation of their potential to develop functional foods and natural ingredients with added value.

Against this background, this study aimed to comparatively characterise the edible pulp of eight native *Inga* species from the Ecuadorian Amazon by determining their physicochemical properties, mineral composition and bioactive compounds, including vitamin C, organic acids, phenolic compounds, carotenoids and chlorophylls, and by evaluating their in vitro antioxidant activity using the DPPH and ABTS assays and their antimicrobial activity against ATCC bacterial and yeast strains. The results provide novel scientific information on these little-studied species and form a basis for future research aimed at utilising these fruits to develop functional foods and products of interest for the food industry.

## 2. Results

### 2.1. Physicochemical Characteristics and Mineral Profile of Inga Species

Table 1 presents the physicochemical characteristics and mineral composition of the edible pulp from eight native *Inga* species collected in the Ecuadorian Amazon. Pulp pH ranged from 5.4 in *I. capitata* to 6.2 in *I. velutina*. Soluble solids showed a wider variation, with the highest concentration recorded in *I. velutina* (24.0 °Brix) and the lowest in *I. marginata* (4.3 °Brix). Titratable acidity ranged from 0.1 to 0.3%, with the highest value observed in *I. marginata*. This species also presented the lowest moisture content (64.5%) and the highest ash content (1.4%), whereas *I. densiflora* had the highest moisture content (86.9%).

The mineral profile showed significant differences between the species assessed. Potassium was the most abundant mineral in all species. *Inga bachryrhachis* presented the highest potassium and calcium concentrations, reaching 2941.3 and 464.9 mg/100 g DW, respectively. The highest iron concentration was recorded in *I. capitata* (66.1 mg/100 g DW), whereas *I. vera* presented the highest sodium concentration (33.4 mg/100 g DW), followed by *Inga leiocalycina* (27.8 mg/100 g DW).

### 2.2. Bioactive Compounds Profiles

Table 2 shows the concentrations of vitamin C, organic acids, major carotenoids and chlorophylls determined in eight native species of the genus *Inga*. The vitamin C content ranged from 1.4 mg/100 g DW (*Inga velutina*) to 13.8 mg/100 g DW (*Inga leiocalycina*).

The organic acid profile also differed among species (*p* < 0.05). *Inga densiflora* presented the highest citric acid concentration (698.9 mg/100 g DW), whereas *I. feuilleei* contained the highest concentrations of malic acid (1480.7 mg/100 g DW) and total organic acids (2233.9 mg/100 g DW). The highest tartaric acid concentration was recorded in *Inga leiocalycina* (882.7 mg/100 g DW). By contrast, *I. bachryrhachis* had the lowest total organic acid concentration (59.3 mg/100 g DW).

β-Carotene was quantified in *I. velutina*, *I. bachryrhachis* and *I. vera*, with the highest concentration observed in *I. vera* (0.8 mg/100 g DW). Lutein was quantified in five species and also reached its highest concentration in *I. vera* (0.3 mg/100 g DW). Consequently, this species presented the highest total carotenoid concentration (1.1 mg/100 g DW). Chlorophyll b was quantified in *I. vera*, *I. feuilleei* and *Inga leiocalycina*, with the highest concentration in *I. vera* (0.9 mg/100 g DW), whereas pheophytin b was quantified only in *I. capitata* (0.2 mg/100 g DW).

Table 3 shows the individual composition and total content of the major phenolic compounds in eight native species of the genus *Inga*. Significant differences (*p* < 0.05) were observed between species for all quantified metabolites, indicating high variability in the accumulation of these bioactive compounds.

*Inga bachryrhachis* also presented the highest concentrations of gallic acid (90.1 mg/100 g DW), 4-hydroxybenzoic acid (391.8 mg/100 g DW), chlorogenic acid (1353.7 mg/100 g DW), caffeic acid (1966.9 mg/100 g DW) and ferulic acid (3015.1 mg/100 g DW). In contrast, the highest syringic acid concentration was recorded in *I. vera* (287.1 mg/100 g DW). Naringenin was quantified only in *I. densiflora* and *I. velutina*, at concentrations of 265.6 and 42.3 mg/100 g DW, respectively.

Rutin, quercetin glucoside and quercetin were quantified exclusively in *I. bachryrhachis*, at concentrations of 420.6, 45.9 and 45.1 mg/100 g DW, respectively. Kaempferol was quantified in *I. capitata* and *I. bachryrhachis*, with respective concentrations of 83.9 and 124.3 mg/100 g DW.

The sum of the major quantified phenolic compounds showed marked variability between species, with values ranging from 123.0 mg/100 g DW (*Inga leiocalycina*) to 7703.7 mg/100 g DW (*Inga bachryrhachis*). *I. capitata* (3064.0 mg/100 g DW) and *I. densiflora* (756.2 mg/100 g DW) also stood out.

### 2.3. In Vitro Antioxidant Activity

Table 4 shows the in vitro antioxidant capacity of the edible pulp from the eight *Inga* species, as determined using the DPPH and ABTS assays. The values obtained differed among species in both assays.

DPPH antioxidant capacity ranged from 0.5 mmol TE/100 g DW in *I. feuilleei* to 4.1 mmol TE/100 g DW in *I. capitata*. The next-highest values were recorded in I. marginata and I. densiflora, at 3.9 and 3.5 mmol TE/100 g DW, respectively.

ABTS antioxidant capacity also reached its highest value in *I. capitata* (5.3 mmol TE/100 g DW), followed by *I. marginata* (5.0 mmol TE/100 g DW) and *I. densiflora* (4.9 mmol TE/100 g DW). The lowest value was recorded in *I. feuilleei* (0.5 mmol TE/100 g DW).

### 2.4. Antimicrobial Activity

Table 5 shows the minimum inhibitory concentrations (MICs) of the freeze-dried hydroethanolic extracts prepared from the edible pulp of the eight *Inga* species against four bacterial and two yeast reference strains.

Six extracts inhibited the growth of *Escherichia coli* across the evaluated concentration range. The lowest MIC was recorded for *I. densiflora* (5.2 mg/mL), followed by *I. bachryrhachis* (20.8 mg/mL), whereas the highest MIC was obtained for *Inga leiocalycina* (125.1 mg/mL). No MIC was established for *I. velutina* or *I. vera* under the experimental conditions.

Only *I. marginata* and *Inga leiocalycina* inhibited *Staphylococcus aureus*, with MICs of 41.9 and 62.6 mg/mL, respectively. Four extracts inhibited *Pseudomonas aeruginosa*. The lowest MIC against this bacterium was recorded for *I. bachryrhachis* (41.7 mg/mL), followed by *I. marginata* (41.9 mg/mL), *I. densiflora* (83.3 mg/mL) and *Inga leiocalycina* (125.1 mg/mL).

*Streptococcus mutans* was the only microorganism inhibited by all eight extracts. The MICs ranged from 5.2 mg/mL for *I. densiflora* to 62.5 mg/mL for both *I. vera* and *I. feuilleei*. None of the extracts inhibited the growth of *Candida albicans* or *Candida tropicalis* within the evaluated concentration range.

The ciprofloxacin and fluconazole positive-control wells showed complete inhibition of visible bacterial and yeast growth, respectively, under the assay conditions.

### 2.5. Statistical Analysis

Figure 1 presents the hierarchical clustering heatmap generated from the standardised species means for the physicochemical, mineral, bioactive compound, antioxidant and antimicrobial variables, together with the transformed antimicrobial activity indices. The analysis revealed differentiated compositional profiles among the eight *Inga* species. *Inga feuilleei* and *Inga leiocalycina* formed the closest pair and were subsequently grouped with *I. vera*. These species showed comparatively low standardised values for several minerals and phenolic compounds, although *I. vera* showed higher standardised values for lutein and chlorophyll b.

A second main cluster comprised *I. capitata*, *I. marginata*, *I. densiflora* and *I. velutina*. Within this cluster, *I. marginata* and *I. velutina* formed the closest pair. *Inga bachryrhachis* formed a differentiated branch and exhibited a markedly distinct compositional profile relative to the other evaluated species.

The profile of *I. bachryrhachis* was characterised by high standardised values for calcium, potassium and several phenolic compounds, including gallic, 4-hydroxybenzoic, chlorogenic, caffeic and ferulic acids, rutin, kaempferol, quercetin glucoside and quercetin. *Inga marginata* showed comparatively high standardised values for titratable acidity and ash, whereas *I. capitata* was characterised by higher standardised values for iron, pheophytin b, and DPPH and ABTS antioxidant capacities. *Inga densiflora* showed comparatively high standardised values for citric acid and naringenin, whilst *I. velutina* was characterised by comparatively high pH and soluble solids values. Overall, the heatmap illustrates the combinations of measured variables that contributed to the differentiation of the species within the present dataset.

Figure 2 presents the PCA of the standardised physicochemical, mineral, bioactive compound and antioxidant variables. The first two principal components explained 62.5% of the total variance, with PC1 and PC2 accounting for 39.1% and 23.3%, respectively.

*Inga bachryrhachis* was positioned in the positive region of PC1 and the negative region of PC2, in the same general direction as potassium and several phenolic compounds, including gallic, caffeic, ferulic, chlorogenic and 4-hydroxybenzoic acids, as well as kaempferol, rutin, quercetin glucoside and quercetin. Calcium also contributed positively to PC1, although its loading was slightly positive on PC2 and therefore did not follow exactly the same directions as *I. bachryrhachis*. Conversely, *I. vera*, *I. feuilleei* and *Inga leiocalycina* were positioned in the negative region of PC1. Among these species, *Inga vera* was projected in the same general direction as tartaric acid, malic acid, lutein and chlorophyll b, whereas *I. feuilleei* and *Inga leiocalycina* occupied closely related positions in the negative PC1-PC2 quadrant.

*Inga marginata* presented the highest positive score on PC2 and was projected in the same general direction as titratable acidity, ash and DPPH antioxidant capacity. *Inga capitata* was positioned in the positive region for both components and was projected towards ABTS antioxidant capacity, iron and pheophytin b. *Inga densiflora* and *I. velutina* were positioned in the negative region of PC1 and the positive region of PC2, in the same general direction as citric acid and naringenin.

## 3. Discussion

### 3.1. Physicochemical Characteristics and Mineral Profile of Inga Species

The physicochemical differences observed among the eight *Inga* species extend the limited compositional evidence currently available for edible fruits of this genus. Pulp pH ranged from 5.4 to 6.2, indicating that the samples were weakly acidic. This range is comparable to the values previously reported for *I. edulis* and *I. insignis*, with pH values of 6.2 and 6.5, respectively. Although interspecific metabolic differences may contribute to this variation, the potential influence of the soil and climatic conditions at each collection site cannot be excluded, as species and geographical origin were not evaluated independently in the present design [7].

The soluble solids content was significantly higher in *Inga velutina* (24.0 °Brix), indicating a greater accumulation of soluble sugars compared with the other species. Soluble solids, such as sugars, are critical indicators of fruit quality and the perception of sweetness. Their accumulation is influenced by metabolic pathways, environmental factors and genetic regulation [8,9].

In contrast, the lower soluble solids content and higher titratable acidity in *I. marginata* could give it a less sweet, more acidic sensory profile. The ratio of soluble solids to acidity is a relevant indicator of acceptability, as it determines the perceived balance between sweetness and acidity. Furthermore, these values are consistent with profiles reported for sweet-fleshed tropical fruits, in which acidity enhances the immediate palatability of fresh fruit [10].

Moisture content ranged from 64.5 to 86.9 per cent, consistent with other studies on Inga edulis and *I. insignis* [7], suggesting that the water content of most species is characteristic of fresh pulp and contributes to their juiciness; however, it may also increase their susceptibility to microbiological spoilage and reduce post-harvest stability [11].

Meanwhile, *I. marginata* exhibited both the lowest moisture content and the highest ash content, indicating a higher relative proportion of dry matter and inorganic components. Potassium was the predominant mineral in all the species evaluated, reaching the highest concentration in *Inga bachryrhachis*. This pattern is consistent with that described by other authors, in which potassium is the main intracellular cation due to its roles in osmotic regulation, photosynthesis, carbohydrate transport, and enzyme activation [12].

The differences observed in calcium and iron demonstrate that each species exhibits a distinct pattern of mineral accumulation. *Inga bachryrhachis* stood out for its high calcium content, whilst *I. capitata* recorded the highest concentration of iron. This variability is consistent with findings from other authors, who indicate that the mineral composition of fruits depends mainly on genetic factors, soil type, nutrient availability, climatic conditions, and the stage of fruit development [13].

### 3.2. Analysis of Bioactive Compounds

The composition of bioactive compounds showed high variability amongst the species assessed, indicating differences in metabolic regulation and in the accumulation of primary and secondary metabolites within the genus *Inga*, as observed in other studies of tropical fruits [7]. The limited information available on the phytochemical composition of *Inga* fruits limits direct comparisons with other species in the same genus.

Vitamin C content differed significantly among species, ranging from 1.4 mg/100 g DW to 13.8 mg/100 g DW. Although these concentrations are considerably lower than those described for tropical fruits that are exceptionally rich in this compound, they are comparable to those reported for other tropical species with moderate ascorbic acid content. Thus, a study evaluating vitamin C in *I. edulis* and *I. insignis* reported concentrations of 6.6 mg/100 g DW and 16.9 mg/100 g DW, respectively [7]. The wide variability observed can be attributed to genetic differences among species, as the biosynthesis and accumulation of vitamin C depend on the genotype, the fruit’s physiological state, and environmental conditions during development [14,15].

Organic acids also exhibited differential behaviour between species. Citric acid predominated in *I. densiflora*, whilst malic acid and tartaric acid reached their highest concentrations in *I. feuilleei* and *Inga leiocalycina*, respectively. Consequently, these latter two species have the highest total organic acid contents. In fruit, the composition of organic acids largely determines flavour, stability, and processing quality, and also influences physiological processes related to respiration and energy metabolism [16,17]. Findings from other researchers studying non-traditional tropical fruits have shown that citric acid is usually the predominant acid; however, some species within the same genus, such as *I. edulis* and *I. insignis*, exhibit a preferential accumulation of specific acids, with the former accumulating citric acid and the latter malic acid, depending on their specific metabolism and the ripening process [7].

In contrast to the high variability observed for organic acids, carotenoids were detected only in a small number of species and at relatively low concentrations. β-Carotene was detected exclusively in *I. bachryrhachis*, *I. vera* and *I. velutina*, whilst lutein was quantified in all species except *I. marginata, I. capitata* and *I. bachryrhachis*. Furthermore, total carotenoids reached their highest concentration in *I. vera*. The low concentrations observed in most of the species assessed suggest that these pigments are not the predominant metabolites in the pulp of these fruits [7,18,19].

During fruit ripening, organic acids such as malic acid and citric acid accumulate in the vacuoles of the pulp cells, contributing to the fruit’s acidity. This acidity usually decreases during ripening, which may affect the appeal of the fresh fruit [9]. Furthermore, malic acid enhances the fruit’s flavour and palatability [10].

The differences observed in chlorophyll content reflect varying degrees of persistence of photosynthetic pigments amongst species. Chlorophyll b was detected in *Inga vera, I. feuilleei* and *Inga leiocalycina,* with the highest concentration recorded in *I. vera* (0.9 mg/100 g DW). Pheophytin b was detected only in *I. capitata* (0.2 mg/100 g DW). These differences may reflect variation in pigment retention or degradation among the analysed fruits; however, pigment metabolism was not directly evaluated. These results are consistent with other studies on *I. edulis* and *I. insignis*, which showed the presence of β-carotene and zeinoxanthin, with total carotenoid concentrations of 3.4 mg/100 g DW and 0.8 mg/100 g DW, respectively [7]. The persistence of these pigments during ripening depends on the balance between chlorophyll degradation and carotenoid synthesis, which determines the characteristic colour change in the fruits [20,21].

The phenolic profile of the eight *Inga* species showed high interspecific variability, both in individual composition and in the sum of the major quantified phenolic compounds. Thus, other authors have noted that phenolic compounds are the main metabolites identified in the genus *Inga* [1]. The sum of the major quantified phenolic compounds ranged from 123.0 mg/100 g DW in *Inga leiocalycina* to 7703.7 mg/100 g DW in *I. bachryrhachis*, whilst *I. capitata* also exhibited a high concentration (3064.0 mg/100 g DW). This heterogeneity suggests that the synthesis and accumulation of phenolic metabolites are strongly influenced by genotype, a situation widely described in tropical fruits and underutilised species, where each species develops specific metabolic pathways that determine its phytochemical composition [7,22,23]. Furthermore, a study on *Inga laurina* demonstrated that this species possesses significant functional potential due to the presence of phenolic compounds [4]; similarly, another study on *I. edulis* and *I. insignis* showed the presence of chlorogenic acid, naringenin, caffeic acid, *m*-coumaric acid, and gallic acid, at concentrations of 1.9 mg/g DW and 3.1 mg/g DW, respectively [7]. Meanwhile, a study on *I. marginata* reported the presence of epicatechin gallate and caffeic acid [6].

Among the hydroxybenzoic acids, *I. bachryrhachis* presented the highest measured concentrations of both gallic acid and 4-hydroxybenzoic acid, reaching 90.1 and 391.8 mg/100 g DW, respectively. Both compounds are metabolites widely distributed in plant tissues and are involved in defence mechanisms against oxidative and biotic stress [24]. In underutilised tropical fruits, gallic acid derivatives have been described as one of the most abundant phenolic families and contribute to the overall antioxidant capacity, although the concentration depends markedly on the species and the physiological state of the fruit [25].

The hydroxycinnamic acid profile differed markedly among the evaluated species. *I. bachryrhachis* exhibited the highest concentrations of chlorogenic acid, caffeic acid and ferulic acid, whilst these compounds were absent or present in low concentrations in other species under the analytical conditions used. This pattern is consistent with studies showing that hydroxycinnamic acids constitute the predominant fraction of phenolic compounds, and that chlorogenic acid is usually the major metabolite, followed by caffeic and ferulic acids [24,25]. The chromatographic profile of *I. bachryrhachis* suggests a comparatively high accumulation of the hydroxycinnamic acids analysed in the pulp. However, metabolite concentrations alone do not demonstrate increased activity of the phenylpropanoid pathway, which is responsible for the biosynthesis of these compounds; these are associated with significant antioxidant capacity and mechanisms of protection against oxidative stress [26,27,28].

As for flavonoids, the distribution was much more specific. Naringenin was quantified only in *I. densiflora* and *I. velutina*, whilst rutin, quercetin and quercetin glucoside were quantified exclusively in *I. bachryrhachis*. Similarly, kaempferol was present only in *I. bachryrhachis* and *I. capitata*. This pattern demonstrates marked metabolic specialisation amongst species within the same genus. Literature reviews indicate that flavonoids such as rutin, quercetin, kaempferol, and naringenin are differentially distributed among plant species and organs due to differences in the genetic regulation of biosynthetic pathways and responses to environmental factors [24].

### 3.3. Antioxidant Activity Analyses

The antioxidant activity of the species assessed showed marked variability among species and between analytical methods, indicating differences in the composition and concentration of reducing compounds present in each fruit. In general, the mean values determined using the ABTS assay were numerically higher than those obtained with the DPPH assay in seven of the eight species. The only exception was *Inga feuilleei*, for which both assays yielded the same mean value of 0.5 mmol TE/100 g DW. This behaviour is consistent with the different reaction mechanisms of the two assays. Whilst DPPH has a greater affinity for lipophilic antioxidants and molecules capable of donating hydrogen atoms, the ABTS radical can react with both hydrophilic and lipophilic compounds via electron- and hydrogen-atom transfer mechanisms. Consequently, both methods tend to yield different results when the samples’ phytochemical profiles are complex; therefore, their combined use enables a more comprehensive assessment of antioxidant capacity [24].

The highest antioxidant activity values were recorded for *Inga capitata, I. marginata* and *I. densiflora* using both methods, whilst *I. feuilleei* consistently exhibited the lowest antioxidant capacity. These results were comparable to a study on *I. marginata*, which reported values between 2.1 and 4.7 mmol ET/100 g DW following microwave extraction, quantified by the DPPH method using different extraction methods and under an experimental design [6]. These results indicate that antioxidant activity does not depend exclusively on the total concentration of a single metabolite group, but rather on interactions among bioactive compounds present in the plant matrix [28]. Furthermore, a study on tropical species from Ecuador demonstrated significant antioxidant activity in *I. edulis* and *I. insignis*, with values of 78.6 µmol TE/g DW and 30.9 µmol TE/g DW, as assessed by the ABTS method, respectively, and values of 7.3 µmol AAE/g DW and 1.5 µmol AAE/g DW, as assessed by the DPPH method. Thus, wide variability in antioxidant activity, as determined by the DPPH and ABTS assays, was attributed mainly to differences in phytochemical composition among species [7]. Furthermore, a study on *Inga laurina* demonstrated that this species has significant functional potential due to its high antioxidant activity, attributable to the presence of phenolic compounds [4].

Although *Inga bachryrhachis* had the highest total phenolic compound content, its antioxidant activity was lower than that observed in *I. capitata* and *I. marginata*. This finding highlights that a higher concentration of total phenols does not necessarily imply greater antioxidant capacity [29]. The observed antioxidant efficacy may have been influenced by the chemical structure of individual compounds, particularly the number and position of hydroxyl groups, the degree of conjugation, and potential synergistic or antagonistic interactions among metabolites [30,31]. Consequently, species with lower phenolic content may exhibit high antioxidant activity when compounds with greater electron-transfer or hydrogen-donating capacity predominate. This phenomenon has been documented in plant matrices and is one of the main reasons why antioxidant activity does not always correlate linearly with total phenolic content [29,32].

Another relevant finding was the low antioxidant activity observed in *I. feuilleei*, despite its appreciable concentrations of vitamin C and malic acid. This confirms that vitamin C, although an efficient antioxidant, is not always the main determinant of the total antioxidant activity of the fruits. Thus, other authors who have studied tropical fruits have shown that phenolic compounds tend to account for a greater proportion of antioxidant activity than ascorbic acid, due to their high capacity to stabilise free radicals and the presence of multiple antioxidant mechanisms of action [33].

### 3.4. Antimicrobial Activity Analyses

The ethanolic extracts of the Inga species exhibited varying levels of antimicrobial activity against the bacteria tested, whilst no species showed any activity against *Candida albicans* or *Candida tropicalis*. The different inhibition patterns may be related to differences in extract composition and microorganism susceptibility. However, because crude hydroethanolic extracts were evaluated, the observed MIC values cannot be attributed to individual compounds or specific interactions among compounds. Thus, plant extracts are complex mixtures of bioactive compounds whose antimicrobial efficacy is determined by the concentration and interactions among phenolic acids, flavonoids, tannins, and other secondary metabolites; consequently, different responses are often observed against Gram-positive and Gram-negative bacteria and yeasts [34].

*Streptococcus mutans* was the only microorganism inhibited by all eight extracts with MIC values ranging from 5.2 mg/mL for *I. densiflora* to 62.5 mg/mL for I. vera and *I. feuilleei*. Six extracts inhibited *Escherichia coli*, with the lowest MIC recorded for *Inga densiflora* (5.2 mg/mL). This high susceptibility is significant because *S. mutans* is one of the main aetiological agents of dental caries, owing to its ability to form biofilms and produce organic acids that demineralise enamel. Thus, other authors who have studied plant extracts have demonstrated that this bacterium is generally more susceptible than other microorganisms to phenolic metabolites, primarily due to disruption of cell membrane integrity and inhibition of biofilm formation. Furthermore, in the case of *E. coli*, this can be attributed to the presence of an outer membrane rich in lipopolysaccharides—a characteristic of Gram-negative bacteria—which acts as an additional barrier hindering the diffusion of numerous phenolic compounds and flavonoids. This behaviour has been extensively described in research on plant extracts, where Gram-negative bacteria typically require higher concentrations to achieve the same inhibitory effect as observed in Gram-positive bacteria [34]. However, other authors demonstrated that the peel and pulp of *I. marginata* exhibited activity at 250 mg/mL against *Staphylococcus aureus* and 500 µg/mL against *Escherichia coli* [6].

In contrast, none of the extracts inhibited the growth of *Candida albicans* or *Candida tropicalis*. The absence of antifungal activity may be explained by structural differences between fungal and bacterial cell walls, as well as by the lower affinity of the compounds in the extracts for fungal membrane components. The absence of measurable inhibition within the evaluated concentration range does not necessarily imply that *Inga* species lack metabolites with antifungal potential. In fact, recent research has identified a peptide derived from a Kunitz-type inhibitor in *Inga laurina* that exhibited potent fungicidal activity against *Candida* species, demonstrating that the genus possesses bioactive molecules with antifungal activity [35].

### 3.5. Statistical Analysis

The hierarchical clustering heatmap enabled the exploratory organisation of the species and measured variables according to similarities in their standardised profiles. The species dendrogram showed two broad clusters. One cluster comprised *I. bachryrhachis, I. vera, I. feuilleei* and *I. leiocalycina*. Within this cluster, *I. feuilleei* and *I. leiocalycina* formed the closest pair and were subsequently grouped with *I. vera*, whereas *I. bachryrhachis* formed a differentiated branch characterised by comparatively high standardised values for potassium, calcium and several quantified phenolic compounds. The second broad cluster comprised *I. capitata*, *I. marginata*, *I. densiflora* and *I. velutina*, with *I. marginata* and *I. velutina* forming the closest pair. These clusters of metabolite co-occurrence and species grouping support the biological interpretation of Inga’s differential profiles. The segregation of the species shows that compositional differences are not gradual, but rather distinct enough to form discrete groups. This finding is consistent with the recognition of certain *Inga* species for their high levels of phenolic compounds [36]. On the opposite branch, the grouping of *I. vera*, *I. feuilleei* and *Inga leiocalycina* reflects their metabolic affinity for organic acids, consistent with the nutritional profile described for Peruvian pacay [37].

The variable dendrogram showed that quercetin, quercetin glucoside, rutin and kaempferol had profiles similar to those of several quantified phenolic acids across the eight species. This pattern may partly reflect shared or interconnected biosynthetic processes; however, biosynthetic pathways were not directly assessed in the present study. [4]. DPPH and ABTS antioxidant capacities were located close to each other in the variable dendrogram, indicating broadly similar patterns across the eight species. Nevertheless, these antioxidant variables were relatively separated from the main group of quantified phenolic compounds. This result indicates that antioxidant capacity did not follow exactly the same interspecific pattern as the individual phenolic compounds included in the analysis [38]. In practical terms, the separation of a phenolic–antimicrobial cluster from one comprising organic acids guides the differentiated use of these species: those in the first group towards functional ingredients with antioxidant and antimicrobial preservative properties, and those in the second towards technological applications based on their acid profile [37].

On the other hand, the first two components accounted for 62.5% of the total variance (PC1 = 39.1%; PC2 = 23.3%). Positive PC1 scores were primarily associated with several quantified phenolic compounds and with potassium and calcium, whereas tartaric acid, malic acid, lutein, chlorophyll *b* and sodium were oriented towards negative PC1 scores. This arrangement reflects contrasting compositional profiles within the present dataset rather than a general separation between all mineral and organic acid variables. This diversification is consistent with the composition described for *Inga*, whose fruits and leaves accumulate derivatives of myricetin and quercetin [4], and in which *I. edulis* and *I. verna* exhibit antioxidant activity whilst *I. marginata* stands out for its high phenolic levels [36]. The covariation of quercetin, rutin, and kaempferol with hydroxycinnamic and benzoic acids replicates the pattern observed in other Amazonian fruits analysed using the same chromatographic methodology, in which quercetin ranks among the most significant differential markers [7].

*Inga bachryrhachis* was positioned towards the phenolic-rich region of the PCA, consistent with its high concentrations of several individually quantified phenolic compounds. In contrast, *I. vera* was projected towards tartaric acid, malic acid, lutein and chlorophyll *b*. *Inga feuilleei* and *I. leiocalycina* occupied closely related positions in the negative PC1–PC2 quadrant. *Inga marginata* showed the highest positive PC2 score and was projected towards titratable acidity, ash and DPPH antioxidant capacity, whereas *I. capitata* was oriented towards ABTS antioxidant capacity, iron and pheophytin *b*. For *I. feuilleei*, the predominance of organic acids is consistent with the nutritional characterisation of the Peruvian pacay, in which these compounds are prominent constituents of the fruit [37]. The isolated position of *I. marginata*, which combines high acidity and antioxidant activity, is consistent with its recognised status as a species rich in phenols and possessing antimicrobial activity [36].

The orientation of DPPH and ABTS along PC2, which diverges from the phenols that dominate PC1, is not anomalous: in multivariate assessments of antioxidant capacity, the assays are distributed amongst different components because they reflect different reaction mechanisms, with ABTS associated with overall capacity and DPPH with more specific scavenging activities [25].

### 3.6. Limitations

This study had limitations, as the fruits were collected from a single geographical area in the province of Pastaza, in the Ecuadorian Amazon, during a sampling period in March. Consequently, the compositional and biological differences observed between the *Inga* species cannot be extrapolated to other geographical locations, harvest periods or environmental conditions.

Antioxidant and antimicrobial activities were assessed exclusively through in vitro assays; therefore, these results should be interpreted as evidence of biological activity under experimental conditions, rather than as direct evidence of in vivo effects. Similarly, antimicrobial activity was determined using crude hydroethanolic extracts, which contain complex mixtures of compounds. Consequently, the observed MIC values cannot be attributed to individual metabolites or specific combinations of compounds without more comprehensive activity-guided fractionation, isolation and characterisation.

Fourthly, chromatographic identification was carried out using RRLC coupled with DAD-UV-Vis detection, by comparing retention times and spectral characteristics with available standards. Whilst this approach allows for the identification and quantification of the target compounds, the absence of mass spectrometry confirmation limits structural characterisation, particularly for compounds with similar chromatographic or UV-Vis characteristics. Future studies incorporating LC–MS or LC–MS/MS would provide greater confidence in compound identification and enable the detection of additional metabolites not included in the present analysis.

Finally, multivariate analyses should be regarded as exploratory. Consequently, clustering patterns and associations between variables are useful for visualising the structure of this dataset.

## 4. Materials and Methods

### 4.1. Fruit Sampling, Physicochemical Characterisation and Mineral Analyses

For each species (Figure 3), three independent biological replicates, each group of 30 fully ripe fruits, were collected in March from different neighbouring trees located in Pastaza Province, Ecuadorian Amazon (1°44′21″ S, 77°29′01″ W). The plant material was taxonomically identified in the herbarium of the Universidad Politécnica Salesiana of Quito, Ecuador and the voucher numbers were QUPS-4764 for *Inga densiflora* Benth.; QUPS-4781 for *Inga marginata* Willd.; QUPS-4786 for *Inga velutina* Willd.; QUPS-4787 for *Inga capitata* Desv.; QUPS-4780 for *Inga bachryrhachis* Harms; QUPS-4765 for *Inga vera* DC.; QUPS-4788 for *Inga feuilleei* DC.; and QUPS-4782 for *Inga leiocalycina* Benth. Fruits showing visible physical damage, symptoms of disease or deterioration were excluded. Sampling was conducted randomly in accordance with the recommendations of the Ecuadorian standard for fresh fruit and vegetable sampling [39]. The three groups were collected and processed independently throughout sample preparation and analysis. Each group was treated as an independent biological replicate and divided into two portions. The first portion was used immediately for physicochemical analyses, whereas the edible pulp from the second portion was frozen at −80 °C and freeze-dried for subsequent determination of minerals, bioactive compounds, antioxidant activity, and antimicrobial activity.

Physical assessments of the fruit pulp were carried out. Subsequently, soluble solids in the pulp were determined using a Hitech RHB-32ATC manual refractometer (Hitech, Chelmsford, MA, USA) [40]; the pH of the liquid fraction obtained after pulping the fruit was measured using a SevenMulti™ potentiometer (Mettler Toledo, Columbus, OH, USA); and total titratable acidity was quantified by titration. Finally, the moisture and ash contents were determined by gravimetric methods using a Memmert BE20 oven (Memmert GmbH+Co. KG, Schwabach, Germany) at 105 °C until constant weight was reached and a Thermolyne muffle furnace (Thermo Fisher Scientific, Waltham, MA, USA) at 525 °C, respectively.

The remaining pulp was frozen at −80 °C and freeze-dried in a Christ Alpha 1-4 LDplus (Martin Christ Gefriertrocknungsanlagen GmbH, Osterode am Harz, Germany). The dried sample was ground and stored in amber vials until analysis. The concentrations of calcium, iron, potassium, and sodium were determined by atomic absorption spectrophotometry, using a Varian Spectra AA-55 instrument (Varian Inc., Palo Alto, CA, USA). Mineral extraction was performed using microwave-assisted acid digestion. Thus, 40 mg of freeze-dried sample was mixed with 5 mL of 65% HNO_3_ in Teflon digestion vessels and processed in a Speed-180 Wave Xpert microwave digestion system (Berghof Products + Instruments GmbH, Eningen unter Achalm, Germany) in accordance with the manufacturer’s digestion programme. After cooling, the digest was diluted to a final volume of 25 mL with deionised water. Calcium, iron, potassium and sodium were quantified at wavelengths of 422.7, 372.0, 404.4 and 589.6 nm, respectively. The calibration curve was prepared using dilutions ranging from 0 to 5 ppm for Ca, 0 to 20 ppm for Fe, 0 to 200 ppm for K, 0 to 10 ppm for Mg and 0 to 8 ppm for Na, starting from a stock solution of 1000 ppm. The calibration curves showed a linear regression model with an R^2^ of 0.999. Mineral analysis was performed independently on the three biological replicates of each species [41].

### 4.2. Analysis of Bioactive Compounds

#### 4.2.1. Bioactive Compound Extractions

Extractions of vitamin C, organic acids, carotenoids, chlorophylls, and phenolic compounds were extracted independently from each of the three biological replicates. For each extraction, 20 mg of freeze-dried pulp from the corresponding biological replicate was used. Thus, three independently prepared extracts were analysed for each species. In all cases, the sample was placed in 2 mL Eppendorf tubes and mixed with the corresponding extraction solvent. The suspensions were then subjected to ultrasound-assisted extraction in a Fisher Scientific FS60D ultrasonic bath (Fisher Scientific, Hampton, NH, USA), and the resulting extracts were separated by centrifugation at 14,000 rpm at 4 °C. Finally, the supernatants were filtered through 0.45 μm PVDF membranes and transferred to vials for chromatographic analysis. Each extract was analysed using three technical chromatographic injections. The triplicate instrumental measurements were averaged to obtain one value for each biological replicate.

For the extraction of vitamin C, 200 μL of 0.2% *DL*-homocysteine and 1200 μL of 3% metaphosphoric acid were used. The mixture was sonicated for 1 min, followed by the addition of 600 μL of deionised water, and then centrifuged for 3 min. Furthermore, the organic acids (citric, malic and tartaric acids) were extracted using 2 mL of 0.02 N sulphuric acid, adjusted with 0.05% metaphosphoric acid and 0.02% homocysteine. Extraction was carried out by sonication for 3 min, followed by centrifugation for 5 min.

For the extraction of carotenoids and chlorophylls, 1 mL of a methanol:acetone:dichloromethane mixture (1:1:2, *v*/*v*/*v*) was used. After 1 min of sonication and 3 min of centrifugation, the coloured phase was recovered, and the solid residue was subjected to successive extractions with 500 μL aliquots of the same solvent system until a completely decoloured residue was obtained. The combined extracts were concentrated on a Buchi TM R-100 rotary evaporator (Fisher Scientific, Hampton, NH, USA) at a temperature below 30 °C, and the dry residue was redissolved in 40 μL of ethyl acetate before chromatographic analysis. Furthermore, the phenolic compounds were extracted using 1 mL of 80% methanol acidified with 0.1% hydrochloric acid. The suspension was homogenised in a VM-300 vortex mixer (Interbiolab Inc., Orlando, FL, USA), sonicated for 2 min, and then centrifuged for 3 min. The solid residue underwent two further extractions with 500 μL of the same acidified solvent, and the resulting extracts were combined before analysis.

#### 4.2.2. Quantification Using Rapid-Resolution Liquid Chromatography (RRLC)

All extracts were analysed using a RRLC 1200 rapid-resolution liquid chromatograph (Agilent Technologies, Santa Clara, CA, USA), coupled with a DAD-UV-Vis detector. The chromatograms were processed using ChemStation software (version 2.15.26) and compared with spectra under the same chromatographic conditions, with retention times recorded; an injection volume of 20 μL was used in all analyses. Where the identification of a peak was uncertain, a known quantity of the corresponding authentic standard was added to the sample and the analysis was repeated. The identification of the peak was considered to be confirmed when the addition resulted in an increase in the area of the original peak at the same retention time without any additional peaks appearing, together with a match between the UV-Vis spectra of the sample peak and the authentic standard.

Quantification was carried out using individual calibration curves of standards and dilutions, with injection volumes of 3, 5, 10, 15 and 20 μL. The calibration curve showed a linear regression model with an R^2^ of 0.999. When the chromatographic response obtained from the initial extraction of 20 mg of freeze-dried pulp was below the limits of detection (LODs), the analysis was repeated using a new aliquot of 40 or 60 mg from the same biological replicate. The same extraction procedure and final extract volume were maintained, resulting in a two- or three-fold enrichment of the analyte in the extract, respectively. The concentration was obtained by interpolating the chromatographic area of the analyte against the calibration curve, and the final concentration was calculated by multiplying the interpolated concentration by the final volume, the dilution factor, and dividing by the dry pulp weight. The results were expressed as mg/100 g dry weight.

Vitamin C was separated on a Zorbax Eclipse XDB-C18 column (4.6 × 50 mm, 1.8 μm; Agilent Technologies) using an isocratic mobile phase consisting of 90% 1.5% monopotassium phosphate in water and 10% 1.8% *n*-acetyl-*n,n,n*-trimethylammonium bromide in HPLC methanol, at a flow rate of 1 mL/min and detection at 244 nm. Quantification was carried out using an *L*-ascorbic acid standard with a concentration range of 0.65–1000 ppb. The method exhibited LODs of 0.20 ppb and quantification (LOQs) of 0.65 ppb.

The organic acids were determined on a YMC-Triart C18 column (150 × 4.6 mm, 3 μm, 12 nm; YMC Europe GmbH, Dinslaken, Germany) using an isocratic mobile phase of 0.027% sulphuric acid at a flow rate of 1 mL/min and detection at 210 nm. Calibration curves were constructed individually for citric, malic and tartaric acids with a concentration range of 0.05–1.00 mg/mL. The method exhibited LODs of 0.06, 0.13, and 0.08 ppm and LOQs of 0.17, 0.39, and 0.26 ppm for tartaric, malic and citric acid, respectively.

Carotenoids and chlorophylls were analysed on a YMC C30 column (150 × 4.6 mm, 3 μm; YMC Europe GmbH, Germany), using methanol (A), methyl-tert-butyl ether (B) and water (C) as the mobile phase, at a constant flow rate of 1 mL/min and with an elution gradient programmed as follows: 95% A and 5% B (0–5 min); 89% A and 11% B (10 min); 75% A and 25% B (16 min); 40% A and 60% B (20 min); 15% A and 85% B (22 min); and 90% A, 5% B and 5% C (25–28 min). Identification was carried out between 350 and 450 nm. Quantification utilised calibration curves of analytical standards of astaxanthin, lutein, violaxanthin, zeaxanthin, trans-β-apo-8-carotenal, α-carotene, β-carotene, zeinoxanthin, β-cryptoxanthin and lycopene. Under the same chromatographic conditions, chlorophyll *a*, chlorophyll b, pheophytin *a* and pheophytin *b* were also determined with a concentration range of 0.01–1.00 mg/mL. The method exhibited LODs of 0.02, 0.029, and 0.003 ppm and LOQs of 0.07, 0.09, and 0.008 ppm for the most important carotenoids, lutein, β-carotene, and zeaxanthin, respectively.

The phenolic compounds were separated using a Zorbax Eclipse Plus C18 column (4.6 × 150 mm, 5 μm; Agilent Technologies) and a mobile phase consisting of a 0.01% aqueous solution of formic acid (A) and acetonitrile (B). Separation was carried out using a gradient starting with 100% A (0 min), followed by 95% A and 5% B (5 min), 50%A and 50% B (20 min), column washing and re-equilibration for 30 min. Detection was performed at 280, 320 and 370 nm. Quantification was carried out using standard curves of gallic acid, 4-hydroxybenzoic acid, caffeic acid, 3-hydroxybenzoic acid, 2,5-dihydroxybenzoic acid, syringic acid, chlorogenic acid, ferulic acid, kaempferol, quercetin glucoside, chrysin, *p*-, *m*- and *o*-coumaric acids, luteolin, naringenin, shikimic acid, vanillic acid, rutin, and quercetin with a concentration range of 0.01–2.00 mg/mL. The method exhibited LODs of 0.009, 0.048, 0.012, 0.006, and 0.007 ppm, and LOQs of 0.028, 0.145, 0.041, 0.014, and 0.021 ppm for the most important phenolics, chlorogenic acid, caffeic acid, ferulic acid, *p*-hydroxybenzoic acid, and gallic acid, respectively. An example of a chromatogram is shown in Figure 4.

### 4.3. Antioxidant Activity Analyses

#### 4.3.1. Extraction for Antioxidant Activity Assays

The extracts intended for the determination of antioxidant activity using the DPPH• and ABTS•+ assays were prepared independently from each of the three biological replicates. For each extraction, 20 mg of lyophilised pulp was mixed with 2 mL of HPLC-grade methanol. The suspension was homogenised on a vortex mixer and subsequently subjected to ultrasound-assisted extraction for 3 min. Upon completion of the extraction, the supernatants were recovered by centrifugation at 14,000 rpm for 3 min at 4 °C and were used for the antioxidant capacity analyses.

As a reference standard, a Trolox stock solution was prepared by dissolving 2 mg of the compound in 2 mL of HPLC-grade methanol. From this solution, the dilutions required to construct the calibration curves for both assays were prepared. Antioxidant activity was expressed in mmol Trolox equivalents per 100 g of dry weight (mmol TE/100 g DW).

#### 4.3.2. Determination of Antioxidant Activity

The DPPH• radical was prepared by dissolving 10 mg of DPPH in 50 mL of HPLC-grade methanol. For the analysis, 20 μL of the extract or the Trolox standard was dispensed into the wells of a 96-well microplate (VWR), followed by the addition of 280 μL of the DPPH• radical solution. HPLC-grade methanol (300 μL) and DPPH• solution (300 μL) were used as blanks. The absorbance of the corresponding sample blank was subtracted from the absorbance of the extract–DPPH reaction before antioxidant capacity was calculated. The microplate was shielded from light with aluminium foil and agitated for 30 min with a microplate shaker. At the end of the incubation period, absorbance was measured at 515 nm using a microplate reader.

The ABTS•+ radical was generated by mixing equal volumes of a 7 mM ABTS solution and a 2.45 mM potassium persulphate solution. The mixture was kept in the dark for 16 h to allow complete radical formation, then diluted to an absorbance of 0.70 ± 0.02 at 734 nm. To determine antioxidant activity, 20 μL of the extract or the Trolox standard was mixed with 280 μL of the ABTS•+ radical solution in a 96-well microplate. The reaction was incubated for 15 min in the dark under constant agitation. Finally, the absorbance was measured at 734 nm using a spectrophotometer with a microplate reader.

### 4.4. Antimicrobial Activity Analyses

#### 4.4.1. Extraction for Antimicrobial Activity Analyses

For each extraction, 2.5 g of freeze-dried pulp was mixed with 25 mL of 50% ethanol. The suspension was homogenised and subsequently subjected to ultrasound-assisted extraction for 6 min. The liquid extract was recovered by centrifugation at 7500 rpm for 5 min at 4 °C. The solid residue was subjected to two further extraction cycles using 25 mL of the same hydroethanolic solution. The supernatants were pooled, filtered through filter paper, and concentrated by evaporation of the ethanol under reduced pressure at temperatures below 40 °C. Finally, the concentrated extract was frozen, freeze-dried and stored frozen until further analysis. Hydroethanolic extracts were prepared independently from each of the three biological replicates per species.

#### 4.4.2. Determination of Antimicrobial Activity

Depending on the amount of freeze-dried extract available for each species, the extracts were reconstituted in 2 mL of sterile distilled water, resulting in initial extract concentrations ranging from 100.0 to 300.3 mg/mL. For the microdilution assay, 100 µL of each extract solution was mixed with 100 of broth, followed by successive twofold serial dilutions in a sterile 96-well microplate with addition of 20 µL of microbial inoculum. Therefore, the final extract concentrations tested were calculated taking into account the initial twofold dilution and the addition of the inoculum, and consequently varied among species.

Antimicrobial activity was evaluated against *Escherichia coli* ATCC 8739, *Staphylococcus aureus* ATCC 6538P, *Pseudomonas aeruginosa* ATCC 9027, and *Streptococcus mutans* ATCC 25175, using a broth microdilution method adapted from the Clinical and Laboratory Standards Institute guidelines [42]. Bacterial suspensions were adjusted to a 0.5 McFarland turbidity standard, and subsequently diluted to obtain final concentrations of approximately 5 × 10^5^ CFU/mL per well. Brain Heart Infusion broth was used for the bacterial assays. *E. coli*, *S. aureus* and *P. aeruginosa* plates were incubated at 37 °C for 24 h, whereas *S. mutans* plates were incubated under CO_2_ enrichment atmosphere. Ciprofloxacin (1.56 mg/mL) was used as a procedural positive inhibition control and appropriate aliquots of these stock solutions were diluted in the corresponding assay medium.

Antifungal activity was evaluated against *Candida albicans* ATCC 10231 and *Candida tropicalis* ATCC 13803, using an adapted broth microdilution procedure. Yeast suspensions were adjusted to a 0.5 McFarland turbidity standard, and subsequently diluted to obtain final concentrations of approximately 2.5 × 10^3^ CFU/mL in each well. Yeast extract peptone dextrose broth was used for the yeast assays, and plates were incubated at 37 °C for 24 h. Fluconazole (1.5 mg/mL) was included as the procedural positive antifungal control and appropriate aliquots of these stock solutions were diluted in the corresponding assay medium.

Each well contained 100 µL of the corresponding extract dilution and 20 µL of microbial inoculum, resulting in a final volume of 120 µL before addition of TTC. Growth, sterility, and positive inhibition controls were included in each assay.

After incubation, 20 µL of 2,3,5-triphenyltetrazolium chloride was added to each well, followed by incubation at 37 °C for 1–2 h in the dark. The minimum inhibitory concentration was defined as the lowest extract concentration at which no visible red formazan formation was observed. As a secondary assessment of recoverable microbial viability, 4 µL from each well was spot-inoculated onto Brain Heart Infusion agar for bacteria, or Sabouraud Dextrose Agar for yeasts, and the plates were incubated at 37 °C for an additional 24 h. The presence or absence of microbial growth was then visually recorded. The MICs were determined independently for each of the three biological extracts prepared for each species. The MIC corresponded to the consensus value obtained in at least two of the three biological replicates. Where no inhibition was observed at the highest final concentration of the extract analysed, the result was reported as >Cmax.

### 4.5. Statistical Analysis

All assays were performed in triplicate (n = 3 biological replicates per species), and the results are expressed as mean ± standard deviation. Each biological replicate consisted of an independent group of 30 fully ripe fruits collected from different neighbouring trees and processed separately. Biological replicates constituted the experimental units used in the statistical analyses. Normality was assessed using the Shapiro–Wilk test, and homogeneity of variance was evaluated using Levene’s test. Differences among *Inga* species were evaluated using one-way ANOVA followed by Tukey’s HSD test when normality and homogeneity assumptions were met; otherwise, the Kruskal–Wallis test followed by Dunn’s multiple-comparison test with Holm adjustment was applied. Statistical significance was established at *p* < 0.05, and different superscript letters indicate significant differences among species for each variable.

For multivariate analyses, non-detected target compounds were coded as zero, indicating non-detection under the analytical conditions. Continuous variables were standardised using Z-scores, and variables with zero variance were excluded. A clustered heatmap was generated from the standardised species means using Euclidean distance and complete-linkage hierarchical clustering. For antimicrobial data included in the heatmap, measured MIC values were transformed into an antimicrobial activity index (1/MIC), such that higher values indicated greater antimicrobial activity. Extract–microorganism combinations showing no inhibition at the highest concentration tested (MIC > Cmax) were assigned an activity index of zero, indicating no detectable antimicrobial activity within the tested concentration range.

Principal component analysis (PCA) was subsequently performed on the standardised dataset to explore the main sources of variation and the relationships among physicochemical characteristics, minerals, bioactive compounds, and antioxidant variables. The biplot shows the eight species and the variables with cos^2^ values ≥ 0.65 on the plane defined by the first two principal components. All analyses were conducted in R version 4.4.1.

## 5. Conclusions

The characterisation of eight native species of the genus *Inga* collected in the Ecuadorian Amazon revealed physicochemical, mineral and bioactive compound profiles that differed markedly between species. Potassium was the predominant mineral, and both the minerals and the organic acids and phenolic compounds exhibited wide interspecific variability, confirming the compositional heterogeneity of the genus and its value as a reservoir of metabolites of interest. Multivariate analysis integrated this variability into a pattern in which the first two dimensions explained 64.5% of the total variance, and hierarchical clustering similarly separated the species according to the relative dominance of two broad compositional profiles, namely a cluster defined by phenolic compounds and minerals, led by *I. bachryrhachis*—the species with the highest sum of the major quantified phenols (7703.7 mg/100 g DW), calcium and potassium—and *I. capitata*, and a cluster characterised by the accumulation of total organic acids, typical of *I. vera*, *I. feuilleei* and *Inga leiocalycina*. It is worth noting that the highest concentration of sum of the major quantified phenols did not correspond to the highest levels of all individual compounds, as *I. bachryrhachis* exhibited the highest values for chlorogenic, caffeic and ferulic acids, kaempferol, quercetin glycoside and quercetin, which demonstrates a distinctive targeted phenolic profile under the analytical conditions used. Antioxidant activity, assessed using the DPPH and ABTS assays, identified *I. capitata*, *I. marginata* and *I. densiflora* as the species with the greatest radical scavenging capacity. With regard to antimicrobial activity, the extracts exhibited a selective spectrum, being more effective against bacteria than against yeasts, as none of the species inhibited the growth of *Candida albicans* or *Candida tropicalis*. The greatest activity was observed against *Streptococcus mutans*, which was inhibited by all species, and against *Escherichia coli*; *I. densiflora* stood out for its high activity against both bacteria (MIC of 5.2 mg/mL), as did *I. marginata*, the only species with activity against all four bacterial pathogens evaluated. These findings provide the first comprehensive characterisation of these underutilised species and lay a scientific foundation for their exploitation as food resources and as raw materials for developing functional foods and natural ingredients.

## Figures and Tables

**Figure 1:**
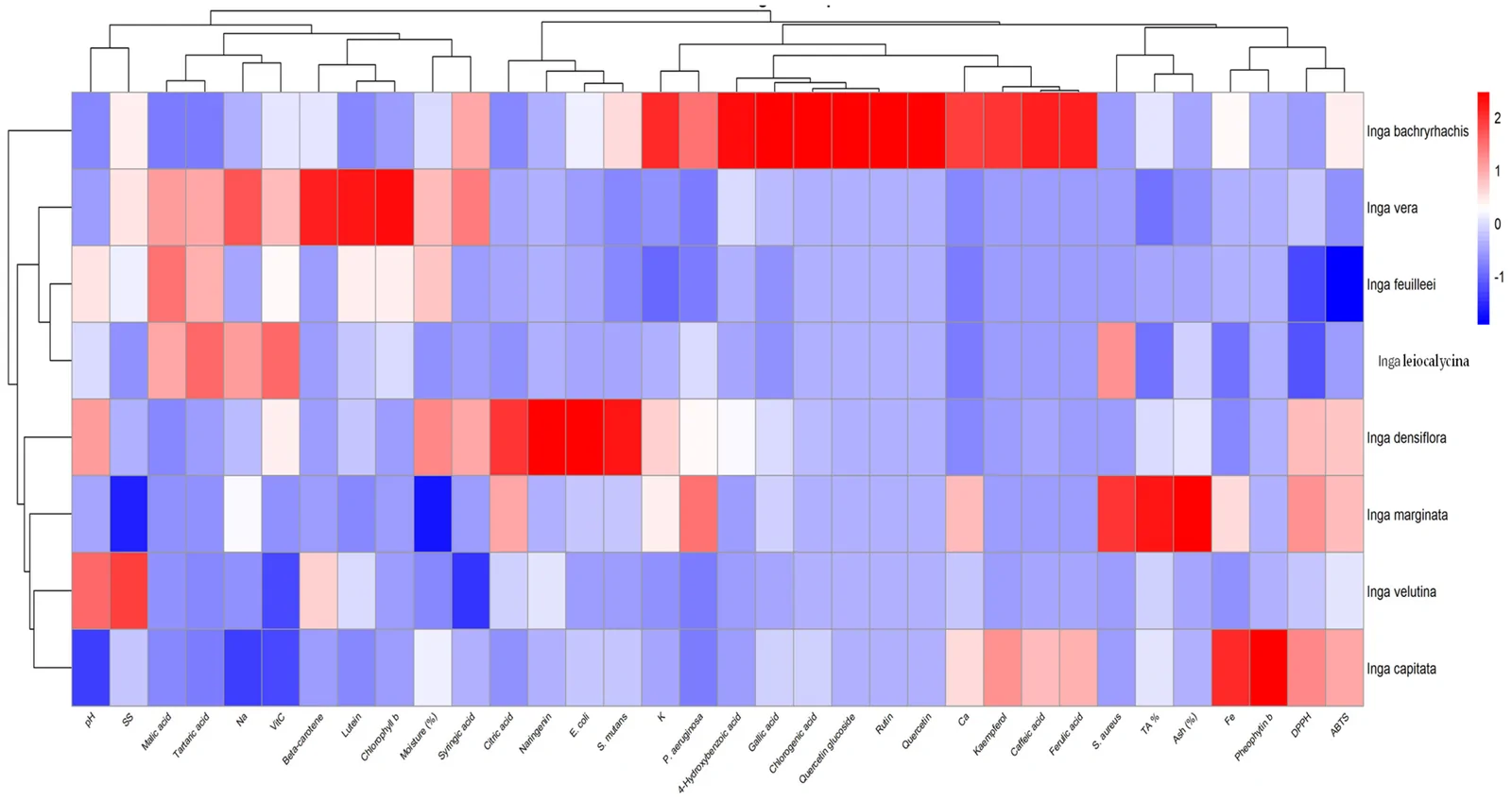
Hierarchical clustering heatmap of the physicochemical characteristics, mineral composition, bioactive compounds, antioxidant capacity and antimicrobial activity response of eight *Inga* species. Red and blue indicate values above and below the mean, respectively. SS, soluble solids; TA, total titratable acidity expressed as citric acid; Vit, vitamin C; Ca, calcium; Fe, iron; K, potassium; Na, sodium; DPPH, 2,2-diphenyl-1-picrylhydrazyl radical scavenging assay; ABTS, 2,2′-azino-bis (3-ethylbenzothiazoline-6-sulfonic acid) radical cation scavenging assay; *E. coli*, *Escherichia coli*; *S. aureus*, *Staphylococcus aureus*; *P. aeruginosa*, *Pseudomonas aeruginosa*; *S. mutans*, *Streptococcus mutans*.

**Figure 2:**
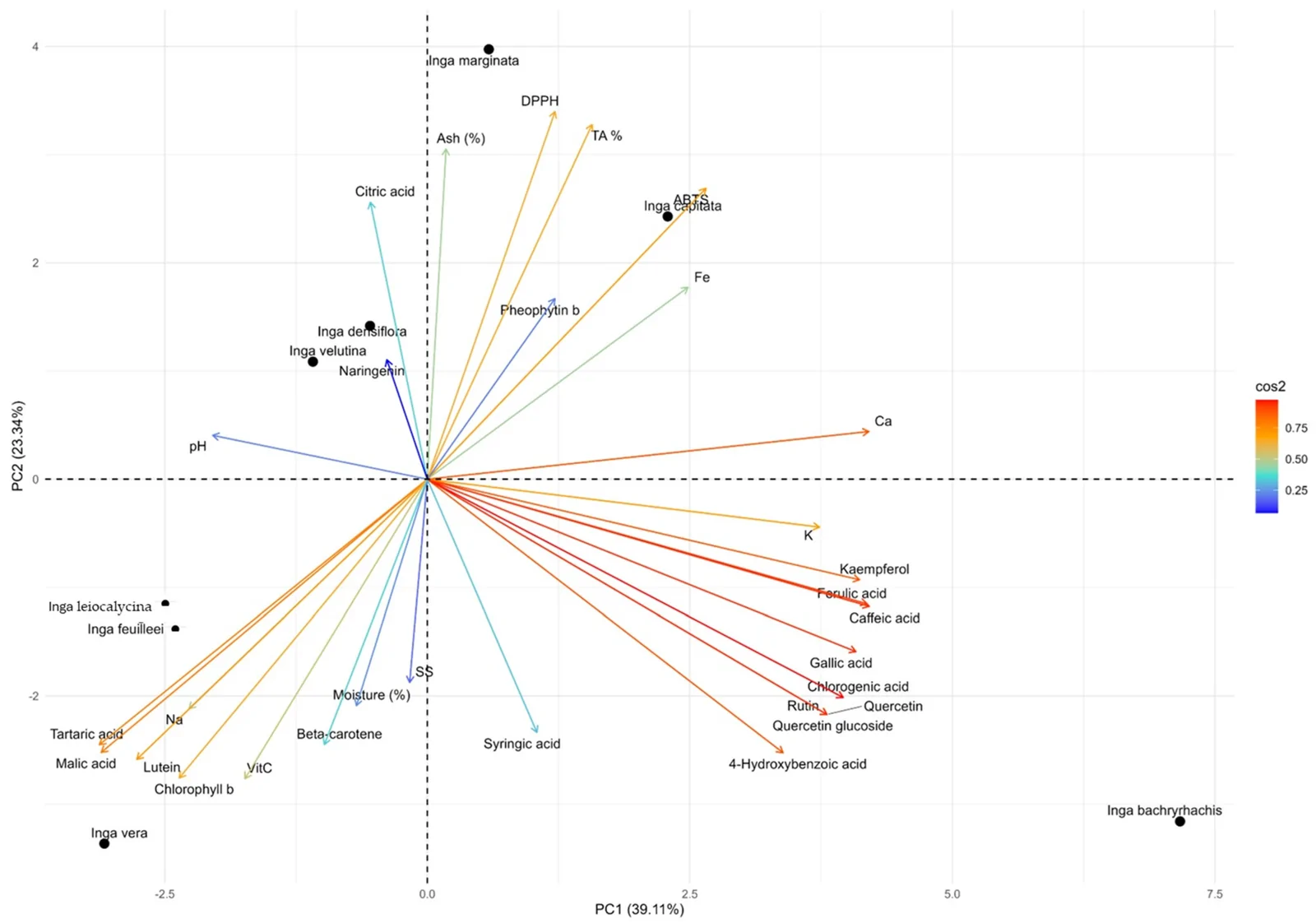
Principal component analysis of the physicochemical characteristics, mineral composition, bioactive compounds and antioxidant capacity of eight *Inga* species. SS, soluble solids; TA, total titratable acidity expressed as citric acid; Vit, vitamin C; Ca, calcium; Fe, iron; K, potassium; Na, sodium; DPPH, 2,2-diphenyl-1-picrylhydrazyl radical scavenging assay; ABTS, 2,2′-azino-bis (3-ethylbenzothiazoline-6-sulfonic acid) radical cation scavenging assay; *E. coli*, *Escherichia coli*; *S. aureus*, *Staphylococcus aureus*; *P. aeruginosa*, *Pseudomonas aeruginosa*; *S. mutans*, *Streptococcus mutans*.

**Figure 3:**
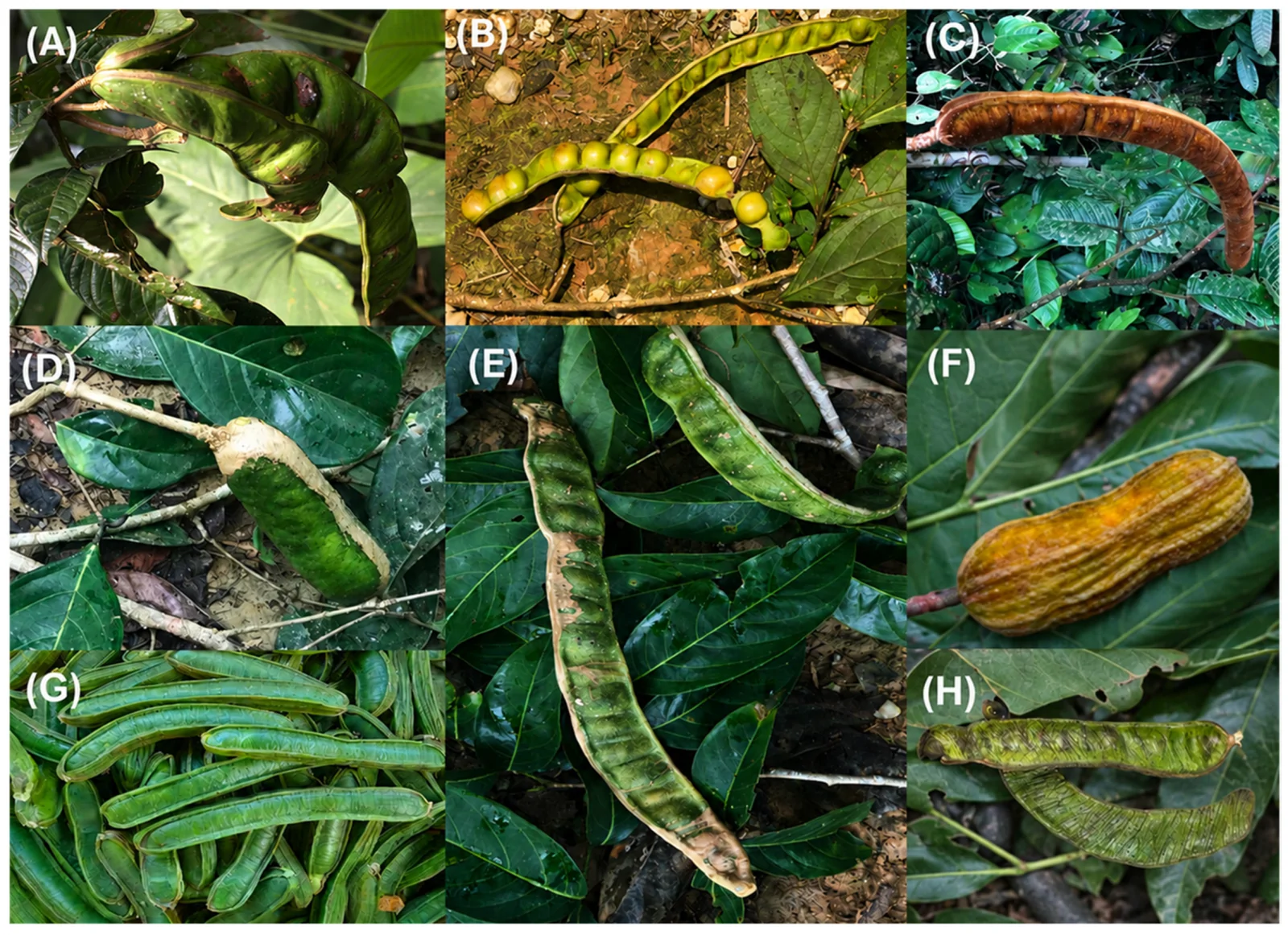
Fruits of the eight *Inga* species evaluated in this study. Note: (**A**), *Inga densiflora* Benth.; (**B**), *Inga marginata* Willd.; (**C**), *Inga velutina* Willd.; (**D**), *Inga capitata* Desv.; (**E**), *Inga bachryrhachis* Harms; (**F**), *Inga vera* DC.; (**G**), *Inga feuilleei* DC.; (**H**), *Inga leiocalycina* Benth.

**Figure 4:**
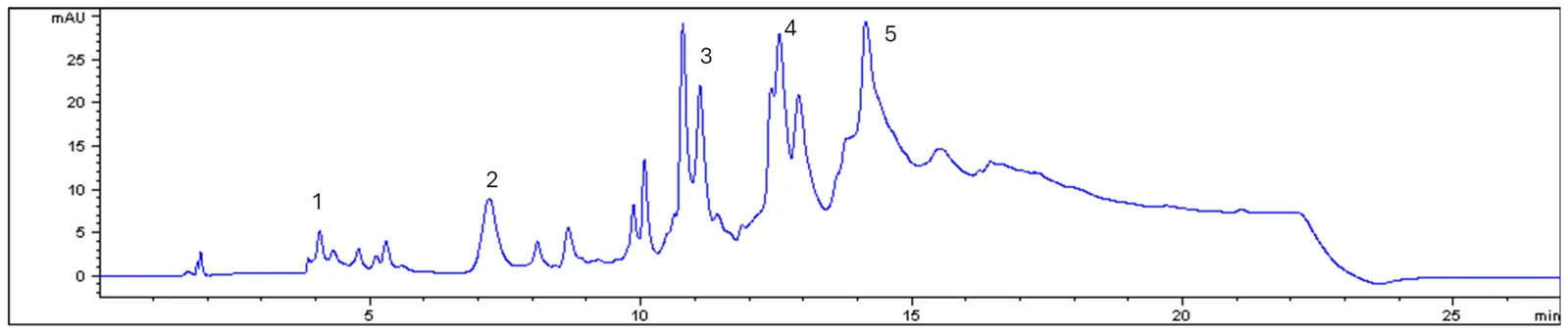
Chromatogram of the phenolic profile of Inga capitata at a wavelength of 280 nm. Note: 1, gallic acid; 2, syringic acid; 3, chlorogenic acid; 4, caffeic acid; 5, ferulic acid.

**Table 1:** Physicochemical characterisation and mineral profile of the edible pulp from eight native *Inga* species collected in the Ecuadorian Amazon.

Parameters	*Inga* *densiflora*	*Inga* *marginata*	*Inga* *velutina*	*Inga* *capitata*	*Inga* *bachryrhachis*	*Inga* *vera*	*Inga* *feuilleei*	*Inga leiocalycina*
pH	6.1 ± 0.1 ^a^	5.6 ± 0.0 ^c^	6.2 ± 0.1 ^a^	5.4 ± 0.1 ^d^	5.6 ± 0.0 ^c^	5.6 ± 0.6 ^c^	5.9 ± 0.1 ^ab^	5.8 ± 0.0 ^b^
SS (°Brix)	11.3 ± 0.5 ^d^	4.3 ± 0.5 ^e^	24.0 ± 2.4 ^a^	12.0 ± 1.2 ^cd^	15.5 ± 1.0 ^b^	16.0 ± 0.0 ^b^	14.2 ± 0.2 ^bc^	10.0 ± 0.0 ^d^
TA (%)	0.1 ± 0.0 ^bc^	0.3 ± 0.1 ^a^	0.1 ± 0.0 ^bc^	0.1 ± 0.0 ^b^	0.1 ± 0.0 ^b^	0.1 ± 0.0 ^c^	0.1 ± 0.0 ^bc^	0.1 ± 0.0 ^c^
Moisture (%)	86.9 ± 1.1 ^a^	64.5 ± 2.6 ^c^	71.4 ± 4.2 ^bc^	78.1 ± 0.7 ^abc^	77.3 ± 2.4 ^abc^	83.3 ± 0.5 ^ab^	83.1 ± 0.4 ^ab^	72.5 ± 17.4 ^abc^
Ash (%)	0.4 ± 0.0 ^b^	1.4 ± 0.3 ^a^	0.2 ± 0.0 ^bc^	0.3 ± 0.0 ^bc^	0.2 ± 0.0 ^bc^	0.2 ± 0.0 ^c^	0.2 ± 0.0 ^bc^	0.4 ± 0.1 ^bc^
Mineral profile (mg/100 g DW)								
Ca	19.9 ± 3.2 ^e^	289.6 ± 45.5 ^b^	116.1 ± 1.6 ^c^	242.3 ± 7.9 ^b^	464.9 ± 86.6 ^a^	28.1 ± 4.0 ^d^	12.2 ± 0.4 ^f^	8.1 ± 0.1 ^g^
Fe	13.3 ± 2.1 ^e^	37.5 ± 0.8 ^b^	15.6 ± 0.2 ^d^	66.1 ± 4.0 ^a^	33.3 ± 0.3 ^c^	20.3 ± 0.2 ^d^	20.5 ± 0.2 ^d^	10.2 ± 0.1 ^f^
K	1728.1 ± 9.2 ^b^	1521.8 ± 31.5 ^c^	588.3 ± 8.0 ^e^	748.4 ± 13.7 ^f^	2941.3 ± 22.8 ^a^	569.9 ± 37.5 ^h^	307.5 ± 3.1 ^h^	847.1 ± 0.8 ^d^
Na	16.2 ± 2.3 ^d^	20.1 ± 1.2 ^c^	13.1 ± 2.1 ^h^	7.4 ± 1.0 ^i^	14.9 ± 0.1 ^f^	33.4 ± 1.7 ^g^	14.4 ± 0.1 ^g^	27.8 ± 2.1 ^b^

Note: Values are expressed as the mean ± standard deviation of three independent biological replicates. SS, soluble solids; TA, total titratable acidity expressed as citric acid; DW, dry weight. Different lowercase letters within the same row indicate significant differences among species.

**Table 2:** Vitamin C, organic acids, carotenoids, chlorophylls and derivatives in the edible pulp of eight native *Inga* species collected in the Ecuadorian Amazon (mg/100 g DW).

	*Inga* *densiflora*	*Inga* *marginata*	*Inga* *velutina*	*Inga* *capitata*	*Inga bachryrhachis*	*Inga* *vera*	*Inga* *feuilleei*	*Inga leiocalycina*
Vitamin C	8.5 ± 0.2 ^c^	4.3 ± 0.2 ^d^	1.4 ± 0.1 ^e^	1.7 ± 0.0 ^e^	7.1 ± 0.3 ^c^	10.5 ± 1.2 ^b^	8.4 ± 0.8 ^c^	13.8 ± 0.9 ^a^
Citric acid	698.9 ± 20.4 ^a^	462.9 ± 31.8 ^b^	172.8 ± 1.4 ^c^	49.9 ± 0.1 ^ef^	22.3 ± 3.1 ^f^	96.2 ± 0.4 ^d^	89.4 ± 2.7 ^de^	54.1 ± 29.7 ^ef^
Malic acid	43.1 ± 0.0 ^d^	126.9 ± 3.5 ^c^	90.1 ± 4.9 ^c^	36.3 ± 0.8 ^d^	30.4 ± 1.2 ^d^	1241.2 ± 13.8 ^b^	1480.7 ± 37.4 ^a^	1213.6 ± 31.8 ^b^
Tartaric acid	81.9 ± 0.1 ^c^	54.1 ± 0.4 ^cd^	32.9 ± 0.3 ^de^	13.0 ± 0.6 ^e^	6.7 ± 0.5 ^e^	685.2 ± 8.4 ^b^	663.8 ± 31.1 ^b^	882.7 ± 13.7 ^a^
Total organic acids	823.8 ± 20.5 ^d^	643.9 ± 28.7 ^e^	295.7 ± 3.8 ^f^	99.1 ± 3.5 ^g^	59.3 ± 3.7 ^g^	2022.5 ± 22.6 ^c^	2233.9 ± 65.8 ^a^	2150.4 ± 15.9 ^b^
β-Carotene			0.4 ± 0.1 ^b^		0.2 ± 0.0 ^c^	0.8 ± 0.0 ^a^		
Lutein	0.1 ± 0.0 ^b^		0.1 ± 0.0 ^b^			0.3 ± 0.1 ^a^	0.1 ± 0.0 ^b^	0.1 ± 0.0 ^b^
Total carotenoids	0.1 ± 0.0 ^c^		0.5 ± 0.0 ^b^		0.2 ± 0.0 ^c^	1.1 ± 0.2 ^a^	0.1 ± 0.0 ^c^	0.1 ± 0.0 ^c^
Chlorophyll b						0.9 ± 0.1 ^a^	0.3 ± 0.0 ^b^	0.1 ± 0.0 ^c^
Pheophytin b				0.2 ± 0.0				

Note: Values are expressed as the mean ± standard deviation. Different lowercase letters within the same row indicate significant differences among species. Blank spaces indicate compounds that were not detected or were below the limit of detection.

**Table 3:** Phenolic compounds individually quantified in the edible pulp of eight native *Inga* species collected in the Ecuadorian Amazon (mg/100 g DW).

	*Inga* *densiflora*	*Inga* *marginata*	*Inga* *velutina*	*Inga* *capitata*	*Inga bachryrhachis*	*Inga* *vera*	*Inga* *feuilleei*	*Inga leiocalycina*
Gallic acid	18.8 ± 1.6 ^b^	16.9 ± 0.5 ^bc^	6.2 ± 0.3 ^cd^	15.8 ± 0.7 ^bc^	90.1 ± 14.0 ^a^	11.7 ± 1.0 ^bcd^	2.6 ± 0.6 ^d^	1.1 ± 0.3 ^d^
4-Hydroxybenzoic acid	109.5 ± 2.4 ^b^				391.8 ± 15.8 ^a^	78.5 ± 6.2 ^c^	25.1 ± 1.8 ^d^	9.7 ± 2.2 ^d^
Syringic acid	252.4 ± 3.3 ^a^	115.3 ± 0.1 ^b^	36.0 ± 2.0 ^c^	132.8 ± 15.5 ^b^	250.2 ± 7.2 ^a^	287.1 ± 38.4 ^a^	117.2 ± 17.6 ^b^	112.3 ± 19.5 ^b^
Chlorogenic acid	35.6 ± 0.3 ^c^		12.5 ± 0.1 ^c^	124.0 ± 12.3 ^b^	1353.7 ± 63.0 ^a^			
Caffeic acid	74.2 ± 4.2 ^c^		33.0 ± 4.5 ^c^	1043.4 ± 106.2 ^b^	1966.9 ± 37.5 ^a^			
Naringenin	265.6 ± 6.5 ^a^		42.3 ± 1.2 ^b^					
Ferulic acid			94.1 ± 0.3 ^c^	1664.0 ± 156.6 ^b^	3015.1 ± 178.3 ^a^			
Rutin					420.6 ± 34.7 ^a^			
Kaempferol				83.9 ± 2.0 ^b^	124.3 ± 8.9 ^a^			
Quercetin glucoside					45.9 ± 0.3 ^a^			
Quercetin					45.1 ± 0.3 ^a^			
Sum of the major quantified phenolics	756.2 ± 13.5 ^c^	132.3 ± 0.5 ^de^	224.0 ± 7.1 ^de^	3064.0 ± 291.9 ^b^	7703.7 ± 25.8 ^a^	377.3 ± 45.7 ^d^	144.9 ± 29.0 ^de^	123.0 ± 22.0 ^e^

Note: Values are expressed as the mean ± standard deviation of three independent biological replicates (n = 3). Different lowercase letters within the same row indicate significant differences among species. The sum of the major quantified phenolics was calculated by adding the concentrations of the individual phenolic compounds determined by RRLC-DAD. Blank spaces indicate compounds that were not detected or were below the limit of detection.

**Table 4:** In vitro antioxidant capacity of the edible pulp from eight native *Inga* species collected in the Ecuadorian Amazon, determined using the DPPH and ABTS assays (mmol TE/100 g DW).

	*Inga* *densiflora*	*Inga* *marginata*	*Inga* *velutina*	*Inga* *capitata*	*Inga* *bachryrhachis*	*Inga* *vera*	*Inga* *feuilleei*	*Inga leiocalycina*
DPPH	3.5 ± 0.2 ^b^	3.9 ± 0.3 ^ab^	1.9 ± 0.2 ^c^	4.1 ± 0.6 ^a^	1.5 ± 0.2 ^d^	2.0 ± 0.1 ^c^	0.5 ± 0.0 ^e^	0.7 ± 0.1 ^e^
ABTS	4.9 ± 1.5 ^a^	5.0 ± 1.6 ^a^	3.7 ± 0.1 ^ab^	5.3 ± 0.8 ^a^	4.3 ± 0.4 ^ab^	2.5 ± 0.3 ^b^	0.5 ± 0.0 ^c^	2.7 ± 0.1 ^b^

Note: Values are expressed as the mean ± standard deviation of three independent biological replicates. Different lowercase letters within the same row indicate significant differences among species.

**Table 5:** Minimum inhibitory concentrations (MICs, mg/mL) of freeze-dried hydroethanolic extracts prepared from the edible pulp of eight native *Inga* species against ATCC reference bacterial and yeast strains.

	Bacterial Strains				Yeast Strains	
	*E. coli* ATCC 8739	*S. aureus* ATCC 6538P	*P. aeruginosa* ATCC 9027	*S. mutans* ATCC 25175	*C. albicans* ATCC 10231	*C. tropicalis* ATCC 13803
*Inga densiflora*	5.2	>83.3	83.3	5.2	>83.3	>83.3
*Inga marginata*	41.9	41.9	41.9	20.7	>41.9	>41.9
*Inga velutina*	>43.3	>43.3	>43.3	43.3	>43.3	>43.3
*Inga capitata*	43.1	>43.1	>43.1	21.5	>43.1	>43.1
*Inga bachryrhachis*	20.8	>41.7	41.7	10.4	>41.7	>41.7
*Inga vera*	>125.0	>125.0	>125.0	62.5	>125.0	>125.0
*Inga feuilleei*	125.0	>125.0	>125.0	62.5	>125.0	>125.0
*Inga* *leiocalycina*	125.0	62.6	125.1	31.3	>125.0	>125.0

## Data Availability

The original contributions presented in this study are included in the article. Further inquiries can be directed to the corresponding author.
